# In Nature's School

**Published:** 1889-08-24

**Authors:** 


### The Doctor's Pulpit.

### IN NATURE'S SCHOOL.

### Ex Nihilo.

Out of nothing nothing can come. Spontaneous generation is an exploded hypothesis. The most searching labours and experiments of the most laborious and minute experimenters have never been able to demonstrate that organic life can spring, as it were *per saltum*, from inorganic matter. Organic life, in ultimate analysis, is cell life. *Omnis cellula e cellula.* Every cell has for its predecessor and parent a pre-existing cell, and a pre-existing cell of its own kind. A diatom does not give birth to a torula, nor a spiral to a rodshaped microbe. Even Bathybius, notwithstanding the watchful soul of Huxley, is now left severely alone in its native mystery. Chaos has no place at all even in the dark cellars and damp dungeons of the world. Cosmos and law are everywhere. Every demonstrable effect has a cause, and a cause fitted to produce that particular effect, and no other. Thanks be to science for making these things so plain that the poorest intellect, trained in the scientific schools, cannot fail to understand them.

But what is the practical significance of this in a practical world? The significance is infinite—in variety, in extent, in importance. "The child is father to the man." True; and the man is father to the child. The knowledge that the man is father to the child in the sense that he transmits to his offspring not only his own features and character, but his own physical constitution, has enabled medical men in thousands and tens of thousands of instances to save children from premature death. That surely is a fact of extraordinary significance! If the casual reader thinks little of it, what thinks the mother whose soul is wrapped up in her little one, and whose last hope may be fixed on the one frail life that lies between her and childlessness? On a lower plane than this may be considered the possibilities of deflected successions to ancient names, to vast possessions, to thrones, and world-wide dominions, which lie trembling in the balance of infant life; and the fact that the scientific certainty of hereditary physical transmission guides the medical man to sure methods of saving and making permanent the flickering fire of vitality on which so many hopes depend.

The true significance of the new medico-scientific knowledge is not understood, because the knowledge itself has not penetrated the popular mind, nor the mind, even, of the educated laity. That is a very great misfortune for the laity, and to the medical profession a very great fault as well as a misfortune. No more striking illustration of the injury which popular ignorance of medical matters can inflict on an honest and highly scientific profession need be sought for than the Maybrick case. In that instance the medical men, guided by their scientific instincts, plainly stated the facts, and stated, also, other facts bearing upon them which made the legal aspects of the case doubtful and difficult. The popular mind loves certainty. "Yes, or no," "black or white," is what it craves for. But many things in Nature and in human life do not admit either of "Yes" or of "No." They are in their essence uncertain, and impossible of clear elucidation. If the facts be uncertain, it is the duty of science to see their uncertainty, and to explain that they are uncertain. That is exactly what the doctors did in the Maybrick case, and what the public, if it had been reasonable and scientific, would have thanked them for doing. But because it was entirely ignorant of the very elements of medical science the public was not able even to understand the medical position, much less to approve it. Therefore it declared medical men to be both ignorant and helpless. Obviously, if medical men wish to be understood and approved by the world when they do well, they must furnish it with the only possible means of forming a sound judgment—viz., with medical knowledge.

But to return. Nothing can come out of nothing. Life cannot come out of not life. Health cannot spring from the absence of health. Strength cannot arise from strengthlessness alone. There must always be an existing life if a new life is to spring from it. There must be a sufficient basis of vitality if a man, weakened by disease or injury, is to regain health; there must be energy enough left in the constitution to convert food, air, and heat into muscular, nervous, and intellectual power if the natural robustness is to be restored. Not only must there be adequate natural vitality in the system itself, but the external objects—food, air, sunlight—on which it is to operate must be present in sufficiency, or health and strength cannot come back again. Nothing is nothing, and can never give rise to something. Before the cannon is fired which is to propel a five hundred pound shot a distance of a mile we must not only have the shot and the powder, but the absolutely indispensable spark. Without the latter the former are mere powerless steel and black granules; without the former the latter is a feeble flicker which the puff of a man's breath will blow out. When the two are brought together a stone fortress may be levelled with the ground or a warship sunk to the bottom of the sea.

What a vast amount of disappointment, disgust, and misery would be spared men if they could learn well this one elementary but all pervading fact that "out of nothing nothing can come." No farmer expects a crop of wheat if he merely ploughs and manures his ground, but fails to put in the seed. As little does he look for a crop of barley if the ground has been sown, not with barley, but with beans. But in matters of happiness and health there are thousands who sow beans and expect a prosperous crop of wheat, and not a few who choke the soil with all manner of weeds, and yet cry out against fortune if there is no yellow corn in harvest time. Few men are pharisaical enough to think themselves superior all round to the majority of their fellows; and yet a man must be an absolute noodle who does not see that the greater part of the misery of the world arises from the fact that the majority of men are such practical blockheads that they expect to reap grapes where they have planted thorns, and to gather figs where they have sown only thistles. Not good morals only, but good sense also is demanded if happiness and health are to be secured.

Health and happiness are the two divinities that everybody desires for his "household gods." Are there any offerings known to science which will keep them constantly present in the household? If there are none which shall ensure their constant presence, there are some which will at least propitiate them. Those offerings consist in—first, an intelligent understanding of the natural laws of health; and, secondly, a cheerful and willing obedience thereunto. If a man will not take the trouble even to understand those elementary laws of health which apply more particularly to himself, he is like a traveller who sets out to explore an unknown country, but who persistently refuses to ask his way. Whilst he who takes the trouble to understand the laws of health, but has not sufficient strength of mind to obey them, is like the drunkard, who knows that he is killing himself with drink, but still persists in drinking. In this world, nothing is given for nothing, nothing comes out of nothing. If a man wishes to draw a just conclusion he must have at least two premisses, and they must be sound ones. If he desires an apple dumpling for dinner he must on no account omit either to prepare the dough or to put in the apples. A man can no more be healthy and happy who neglects the laws that conduce to health and happiness than he can keep his skin dry if he persists in standing uncovered in a deluge of midsummer rain.

